# Cortical circuit-based lossless neural integrator for perceptual decision-making: A computational modeling study

**DOI:** 10.3389/fncom.2022.979830

**Published:** 2022-11-03

**Authors:** Jung Hoon Lee, Joji Tsunada, Sujith Vijayan, Yale E. Cohen

**Affiliations:** ^1^Allen Institute for Brain Science, Seattle, WA, United States; ^2^Department of Otorhinolaryngology, Perelman School of Medicine at the University of Pennsylvania, Philadelphia, PA, United States; ^3^School of Neuroscience, Virginia Tech, Blacksburg, VA, United States; ^4^Department of Neuroscience, University of Pennsylvania, Philadelphia, PA, United States; ^5^Department of Bioengineering, University of Pennsylvania, Philadelphia, PA, United States

**Keywords:** lossless integrator, inhibitory cell types, perceptual decision-making, cortical circuits, computational model

## Abstract

The intrinsic uncertainty of sensory information (i.e., evidence) does not necessarily deter an observer from making a reliable decision. Indeed, uncertainty can be reduced by integrating (accumulating) incoming sensory evidence. It is widely thought that this accumulation is instantiated *via* recurrent rate-code neural networks. Yet, these networks do not fully explain important aspects of perceptual decision-making, such as a subject’s ability to retain accumulated evidence during temporal gaps in the sensory evidence. Here, we utilized computational models to show that cortical circuits can switch flexibly between “retention” and “integration” modes during perceptual decision-making. Further, we found that, depending on how the sensory evidence was readout, we could simulate “stepping” and “ramping” activity patterns, which may be analogous to those seen in different studies of decision-making in the primate parietal cortex. This finding may reconcile these previous empirical studies because it suggests these two activity patterns emerge from the same mechanism.

## Introduction

One of the fundamental operations of the brain is to transform representations of external sensory stimuli (i.e., sensory evidence) into a categorical judgment, despite the inherent uncertainty of this sensory evidence. For instance, we can determine the direction of the wind, even though its instantaneous direction continuously fluctuates. It is widely thought that this moment-by-moment uncertainty is minimized by temporally integrating (accumulating) this incoming sensory evidence (Roitman and Shadlen, 2002; Smith and Ratcliff, 2004; Gold and Shadlen, 2007; Goldman et al., 2009). Notably, drift diffusion model has shown that noisy integration of evidence could explain various experimental observations such as speed-accuracy trade-off regarding the decision-making (see Ratcliff et al., 2016) for a review. Potential neural correlates of this accumulation process have been identified in a variety of brain areas, including the lateral intraparietal cortex (area LIP) (Roitman and Shadlen, 2002; Mazurek et al., 2003; Gold and Shadlen, 2007), the prefrontal cortex (Kim and Shadlen, 1999), and the frontal eye fields (Ding and Gold, 2012). In particular, spiking activity in these brain areas appears to smoothly “ramp up” (accumulate; i.e., linearly increasing activity over time) prior to a perceptual decision. Further, the rate of this accumulation, which governs the time to reach a decision threshold (i.e., the time to the perceptual decision), is correlated with the ambiguity of the sensory evidence: as the evidence becomes less ambiguous (e.g., the instantaneous fluctuations in wind direction decrease), the rate of the ramping increases (Gold and Shadlen, 2007).

Such neural integration has been modeled in two very different ways, each of which relies on different coding strategies and mechanisms of integration (Goldman et al., 2009). In the first type of model, rate-code neural integrators (NI) integrate sensory evidence and represent accumulated evidence as monotonically increasing (“ramping”) spiking activity. In this rate-code model, the firing rates of individual neurons increase over time in response to continuous inputs (Roitman and Shadlen, 2002; Gold and Shadlen, 2007; Wang, 2012). In an alternative model, location-code NIs store accumulated evidence as the location of highly elevated spiking activity. In such a location-code NI, the location of these highly active neurons, which are referred to as a “bump,” travels through a network over time (Skaggs et al., 1995; Song and Wang, 2005). That is, the location of bump activity corresponds to the total amount of accumulated evidence.

Because ramping activity has been found in several studies of perceptual decision-making (Gold and Shadlen, 2007; Goldman et al., 2009), it is generally believed that a rate-code NI is the natural circuit candidate for neural integration of sensory information. In the rate-code NI, recurrent excitatory currents compensate for the leak currents, allowing excitatory neurons to integrate external sensory inputs (supporting a choice). We note that this rate-code NI has two distinct properties. First, its dynamics strongly depends on the relationships between the leak and recurrent currents. When the recurrent currents are precisely balanced with the leak currents, the rate-code NI would become a lossless NI, which can perfectly integrate sensory evidence and retain the evidence during the temporal gap of the external evidence. When the recurrent currents are stronger or weaker than the leak currents, the rate-code NI would overestimate or underestimate the evidence. Earlier studies (Kiani et al., 2013; Liu et al., 2015) suggested that the brain may utilize lossless integrators, suggesting that the recurrent currents in the rate-code NI need to be precisely tuned to compensate for the leak currents. Given the stochastic nature of neural systems, the perfect tuning would be hard to accomplish (Kiani et al., 2013). Notably, the location-code NI can readily account for the lossless integration (Song and Wang, 2005). Second, all neurons in the rate-code NI show homogenous behaviors. During integration, all neurons’ responses would ramp. That is, the rate-code NI cannot natively explain “stepping activity” recently identified during decision-making (Latimer et al., 2015).

Based on the fact that the location-code NI can readily explain the lossless integrator, we hypothesized that the location-code NI can support perceptual decision-making. To address this hypothesis, we asked two questions. First, can a cortical circuit support the location-code NI? Using a computational model, we found that a neural circuit consisting of two major inhibitory neuron types and depressing synapses can create bump activity, traveling during the presence of sensory evidence but staying at the same location during the temporal gap in the flow of sensory evidence. That is, this circuit can serve as a lossless NI. Second, what kind of predictions can the newly proposed NI make? We found that an independent population of “readout” neurons could convert evidence stored in the NI to population ramping activity experimentally observed when they are connected with one another *via* recurrent connections. Interestingly, while the population activity monotonically increased, the individual neurons’ responses show diverse patterns similar to stepping or ramping activities.

These results raised the possibility that the same mechanisms could underlie both stepping and ramping activities. Although this prediction is purely derived from computational models, we believe that it could aid future studies on perceptual decision-making. *To the best of our knowledge*, there is no direct evidence supporting location-code NIs associated with perceptual decision-making, but sequential activations of neurons, consistent with bump activity propagation, have been reported in multiple brain regions (Ikegaya et al., 2004; Tang et al., 2008; Pulvermuller and Shtyrov, 2009; Harvey et al., 2012; Xu et al., 2012). In the future, we will study the properties of the newly proposed location-code NI and test its predictions against experimental data.

## Results

**FIGURE 1:**
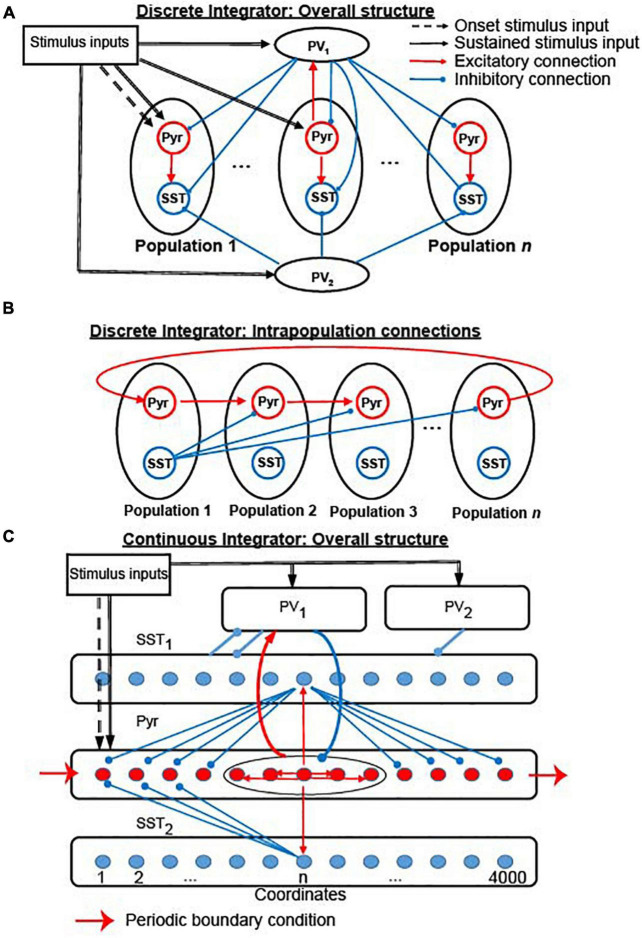
The structure of the two versions of our integrator. **(A)** Connectivity between all 19 neuronal populations in the *discrete* integrator. **(B)** Interconnectivity between the 17 Pyr-SST populations; see section “Materials and methods” and Tables 1, 2 for more details and parameters. Red and blue arrows indicate excitatory and inhibitory connections within the network model, respectively. Dashed and thick black arrows represent onset and sustained stimulus inputs, respectively. **(C)** Structure of *continuous* integrator. The five neuronal populations (Pyr, PV_1_, PV_2_, SST_1_, and SST_2_) interact with each other *via* connections shown in the figure. The thin red arrows and blue arrows represent the excitatory and inhibitory connections between individual neurons, respectively. In contrast, the thick arrows (including red and blue) show connections between the neuronal populations. All connections between populations are randomly established. Sensory inputs are introduced to Pyr, PV_1_, and PV_2_ (dashed arrows). Periodic boundary condition is used to connect Pyr cells, as shown in the red arrow; see section “Materials and methods” and Table 3 for more details and parameters.

This section describes how cortical circuits can implement a lossless integrator. In section “Stability of the rate-code neural integrators,” we examine the stability of the rate-code NI during the temporal gap. Section “Cortical circuits that can support location-code neural integrators” describes simulation results suggesting that generic cortical circuits (Figure 1A), which contain two common types of inhibitory neurons (Beierlein et al., 2003; Rudy et al., 2011) and depressing synapses (York and van Rossum, 2009; Romani and Tsodyks, 2015), can readily realize a lossless (“perfect”) location-code NI. In section “Continuous location-code neural integrator,” we propose a location-code NI that can have continuous attractors (Figure 1C). Finally, in section “Potential links to decision-making: the contribution of elective and exclusive connections between integrators and readout neurons,” we discuss how evidence accumulated in our integrators can be converted to decision-related neural responses (decision variables). Interestingly, this readout activity maps onto two different modes of spiking activity that have been identified during neurophysiological studies of decision-making: classic “ramping” activity (Roitman and Shadlen, 2002) and newly identified “stepping” activity (Latimer et al., 2015).

### Cortical circuits can readily implement lossless location integrator

#### Stability of the rate-code neural integrators

**FIGURE 2:**
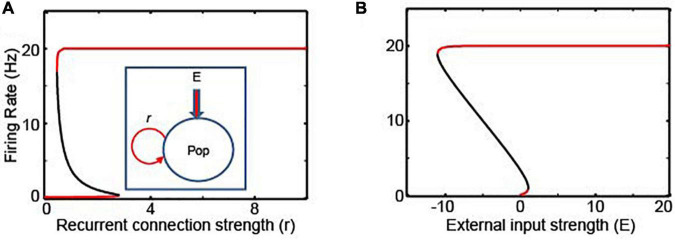
The bifurcation analysis of rate- and location-code NIs. **(A,B)** Bifurcation analyses with the recurrent connections (*r*) and the external inputs (*E*) as bifurcation parameters for the recurrent rate-code network model, respectively; the schematics this network model is shown in the inset of panel **(A)**. Red and black lines represent stable and unstable steady solutions, respectively. Pop in the figure denotes a neuronal population.

We first evaluated the stability of the rate-code NI using the firing rate model. A rate-code NI was modeled with a single recurrent population (Goldman et al., 2009) (Equation 1; see the inset of Figure 2A).

The firing rate of the rate-code recurrent network obeys Equation 1 (Goldman et al., 2009):


(1) $$\tau_{m}\,\frac{d\,F_{e}}{d\,t}=-F_{e}+F_{m\,a\,x}\,\frac{1}{\left[1+e^{-\beta \,\left(r\,F_{e}+E-\theta \right)}\right]}$$

where, *F*_*e*_ and *r* are the firing rate and recurrent connections, respectively; *F*_*max*_ is the maximum firing rate; θ is the spiking threshold; *E* is the external input; and β represents the strength of stochastic inputs (Ermentrout and David, 2010). The first term in the right-hand side of Equation 1 represents the leak current, which corresponds to the subthreshold dynamics of leaky integrate-and-fire neurons (Miller and Fumarola, 2012). The selected default parameters are *F*_*max*_ = 20, β = 1, θ = 0.5, *r* = 1, and *E* = 0, unless stated otherwise. We modeled the gain (transfer function; i.e., the number of spikes that a neuron can generate in response to afferent synaptic activity) with a logistic function (Ermentrout and David, 2010); the firing rate of this neuron is not zero even when the sum of its synaptic inputs is smaller than the spiking threshold.

We tested the stability of this network by conducting a bifurcation analysis with the XPPAUT analysis platform (Ermentrout, 2007). A bifurcation analysis identifies the steady-state solutions, in which a system can stay indefinitely until perturbed. Moreover, this analysis clarifies whether the steady-state solutions are stable in response to the perturbations of bifurcation parameters (which, in our analysis, is the strength of the recurrent connections *r* and the external inputs *E*; see the inset of Figure 2A). In Figures 2A,B, the stable and unstable steady-state solutions are shown in red and black, respectively. As seen in these figures, this recurrent rate-code network (Equation 1) has only two stable attractor states, in which neurons either fire at their maximum rate (*F*_*max*_) or become quiescent. This implies that if there is a small perturbation in the strength of the recurrent connections or if there are changes in the external sensory inputs (e.g., a temporal gap in the incoming sensory information, *E* = 0), this network could lose temporally accumulated information (Kiani et al., 2013).

#### Cortical circuits that can support location-code neural integrators

Cortical circuits have three common properties that are relevant for our model. First, pyramidal (Pyr) neurons in sensory cortex are topographically organized as a function of their sensory response profiles *via* spatial (Hubel and Wiesel, 1962, 1968) and functional (Ko et al., 2013) connections. Second, cortical circuits also contain parvalbumin positive (PV) and somatostatin positive (SST) inhibitory interneurons (Rudy et al., 2011). PV neurons have a fast-spiking pattern of activity, whereas SST neurons have a low-threshold spiking pattern. For our purposes, it is important to note that, although most inhibitory interneurons are broadly tuned to sensory inputs, the response profiles of SST neurons can be as sharply tuned as those of Pyr neurons (Ma et al., 2010). Third, *via* lateral inhibition, SST neurons inhibit neighboring cortical neurons (Markram et al., 2004; Adesnik et al., 2012; Zhang et al., 2014; Jiang et al., 2015).

Based on previous modeling studies (York and van Rossum, 2009; Romani and Tsodyks, 2015) that proposed propagating bump activity can be elicited by depressing synapses, we built a cortical network model (Figure 1A), in which Pyr neurons interacted with one another through intra-population depressing synapses (Markram et al., 1998; Reyes et al., 1998; Fuhrmann et al., 2002; Petersen, 2002; Cheetham and Fox, 2010; Lefort and Petersen, 2017) and inter-population unidirectional static synapses. We refer to this cortical network model as the “discrete” integrator; see section “Materials and methods” for more details. Transient sensory stimuli (100 ms), which mimicked sensory-driven onset responses in sensory cortex (Cleland et al., 1971; De Valois et al., 2000; de la Rocha et al., 2008; Piscopo et al., 2013), only drove Pyr cells in the first population. In contrast, sustained sensory stimuli (after 100 ms) drove Pyr neurons in all neuronal populations. In our first simulation, we only provided Pyr and PV neurons with sensory evidence at two discrete time intervals: time = 100–300 ms and during time = 800–1,000 ms.

**FIGURE 3:**
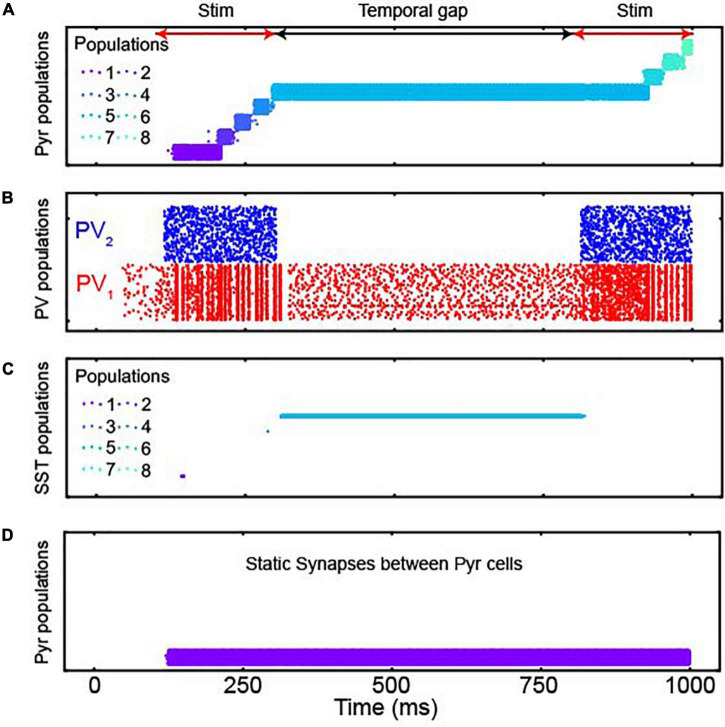
The responses of populations of the discrete integrator. **(A)** Spiking activity of Pyr neurons in all 17 neuronal populations; each population had 400 Pyr neurons. Each row in the plot shows the spike times of an individual Pyr neuron. Each of the 8 populations are shown in different colors; see legend for the color codes of a subset of these populations. Although the model contains 17 populations, only 8 populations were activated during our simulations, which we display here. The red and black arrows show sensory-stimulus periods and the temporal gap between them, respectively. **(B)** PV_1_ and PV_2_ activity during the sensory-stimulus periods and the temporal gap between both. Both PV populations contained 1,088 PV neurons. **(C)** SST neuron activity in all 8 populations; there are 16 SST neurons in each population. The same color scheme is used as in panel **(A)**, and during the temporal gap, active SST and Pyr neurons have the same color, indicating that active SST and Pyr neurons belong to the same population. **(D)** Pyr activity when all depressing synapses are replaced with static ones.

As seen in Figure 3A, the Pyr populations were sequentially activated by sensory stimulation. Further, on average, both populations of PV neurons were more active during sensory stimulation than during the temporal gap (Figure 3B). More importantly, when there was a temporal gap in the sensory evidence (as indicated by the black double-headed arrow in Figure 3A), the sequential activation of the network stopped but activity was maintained by a specific population of Pyr neurons (Pyr population 5 in Figure 3A). That is, during a temporal gap in the sensory evidence, the network retained the accumulated information, a finding that is consistent with lossless integration. When we presented the second sensory stimulus, information resumed propagating through the network as seen by the sequential activation of Pyr population 6, followed by population 7, etc.

When we explored the network in more detail, we found key roles for the inhibitory neurons and for the depressing synapses. For example, SST neurons were active only during the temporal gap (Figure 3C) and that bump activity did not propagate when we replaced the depressing synapses with static synapses (Figure 3D). We also noted that the non-specific feedback inhibition of PV_1_ neurons play a key role to activate an appropriate population of neurons (i.e., Pyr population 6 in Figure 3A, following the temporal gap). Without this inhibition, when we presented the second sensory stimulus, Pyr population 1 (which was activated by the first initial 100-ms of sensory stimulation) was inappropriately activated. This altered the amount of accumulated information (Supplementary Figure 1).

### Continuous location-code neural integrator

The discrete location-code NI (Figure 1A) has limited precision: the accumulated evidence needs to be quantized to be stored in the discrete populations. This limitation, however, is not a fundamental restriction because this discrete network can be generalized to have continuous attractor states by distributing Pyr and SST neurons into circular lattices with uniquely assigned coordinates (Figure 1C). We call this a “continuous lossless integrator.” For convenience, we refer to the direction from lower to higher coordinates as the clockwise direction and higher to lower as counterclockwise. Two Pyr neurons were connected in this network if the difference between their coordinates was ≤200. Because the connections were symmetrical, each Pyr neuron made excitatory synapses with 400 of its neighboring Pyr neurons.

All Pyr and SST neurons formed non-specific connections with PV_1_ neurons. PV_2_ neurons exclusively provided feedforward inhibition to SST_1_ neurons. The connections between Pyr neurons and SST neurons were formed based on their coordinates in the circular lattice. (1) Pyr neurons made one-to-one synaptic (“topographic”) connections with SST_1_ and SST_2_ neurons, when they had the same coordinates. (2) A SST_1_ neuron inhibited a Pyr neuron when the (absolute) difference between their coordinates was ≥200. (3) A SST_2_ neuron inhibited a Pyr neuron when the coordinate of a Pyr neuron was lower than that of a SST_2_ neuron and when the (absolute) coordinate difference was between 400 and 800. Because of this connectivity pattern, the propagation of bump activity in the counter-clockwise direction was dampened, which is possible with symmetrical chain-like recurrent connections, and only bump activity in the clockwise direction propagated through the network.

**FIGURE 4:**
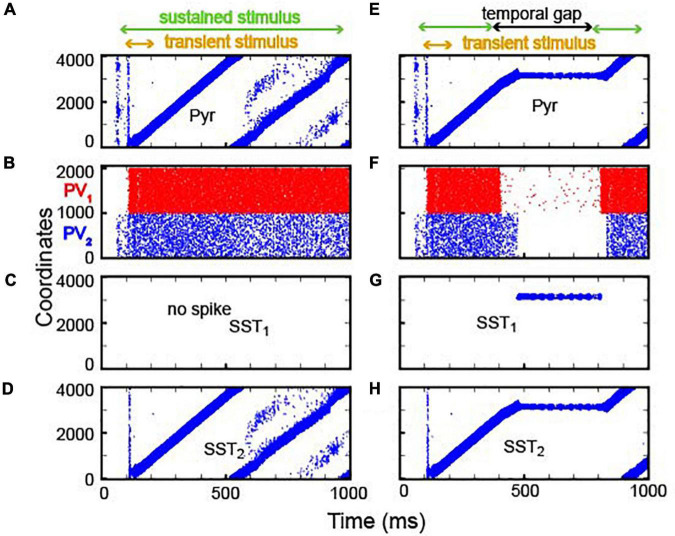
Integration of sensory inputs with and without temporal gaps. **(A–D)** Spiking activity in Pyr, PV (PV_1_ and PV_2_), SST_1_ and SST_2_ neurons in response to constant sensory input. The model received two types of sensory inputs (the onset inputs marked by yellow arrows and the sustained inputs marked by greed arrows). The onset inputs are introduced to 400 neurons simultaneously, and the sustained inputs are introduced to all neurons. During stimulus presentation (100–1,000 ms, marked as the green arrow), the location of bump propagates through the circular lattice: PV neurons fire asynchronously. SST_1_ neurons (shown in panels **C**,**G**) are quiescent, whereas SST_2_ activity (shown in panels **D,H**) mimics Pyr activity. **(E–H)** Raster plots of Pyr, PV, SST_1_, and SST_2_ activity, respectively, when there was a temporal gap between stimulus presentations. During the gap (300–800 ms, marked by the black arrow), SST_1_ neurons became active **(G)**, and the bump activity of Pyr neurons stayed at the same location.

In our first analysis, we examined whether our continuous integrator could integrate sensory evidence (see Table 3 and Supplementary Figure 2 for model-parameter details). To test this integrator, we first presented a transient sensory input (time = 100–200 ms) to the first 400 Pyr neurons (i.e., those with the lowest coordinates), followed by a more sustained sensory stimulus (time = 100–1,000) to all Pyr and PV neurons. As seen in Figure 4A, this transient sensory stimulus elevated the rate of spiking activity strongly enough to generate bump activity. However, once generated, the feedback inhibition mediated by the PV_1_ neurons was strong enough to prevent all other excitatory neurons from spiking during the presentation of this transient sensory stimulus.

After the offset of this transient input, bump activity propagated to other Pyr neurons in the clockwise direction (Figure 4A). Due to the periodic boundary condition, bump activity repeatedly circulated the integrator. In our model, because excitatory synapses had not fully recovered, when the bump activity returned to the initial location, it dissipated. As a consequence, the non-specific inhibition mediated by PV_1_ neurons became weaker, which, in turn, resulted in Pyr activity at multiple locations (see Pyr cell activity after 500 ms in Figure 4A). Concurrently, PV_1_ and PV_2_ neurons fired asynchronously (Figure 4B). SST_1_ neurons were quiescent (Figure 4C), but SST_2_ neurons, which received excitation from Pyr *via* topographic connections, mimicked Pyr activity (Figure 4D). This SST_2_ activity prevented bump activity from propagating in the counterclockwise direction due to its asymmetrical feedback inhibition onto Pyr neurons.

Next, we tested whether this network could perform lossless integration. Like the discrete neural integrator, we presented two epochs of sensory stimuli (time = 100 and 300 ms and time = 800–1,000 ms) that were separated by a period without sensory stimulation. For simplicity, we did not consider the onset input at 800 ms because this input had no impact on the network dynamics in the discrete integrator (Figure 3A and Supplementary Figure 1). As seen in Figure 4E, bump activity cascaded through the network until there was a temporal gap in the sensory evidence. During the temporal gap, bump activity remained in the same location. Then, it resumed moving from the previous location, as information was reintroduced, consistent with lossless integration.

As in the discrete integrator, during the temporal gap in sensory information, the PV_1_ and PV_2_ neurons (Figure 4F) became quiescent. As a result, the inhibition from the PV_1_ and PV_2_ neurons to the SST_1_ neurons was reduced, which, thereby, increased SST_1_ activity (Figure 4G). The firing pattern of SST_2_ neurons was comparable to that of the Pyr neurons (Figure 4H). Because the SST_1_ neurons were topographically connected to Pyr neurons, the SST_1_ inhibited non-active Pyr neurons, which prevented bump activity from propagating to a new location. Together, this transforms the network into a quasi-stable attractor network.

**FIGURE 5:**
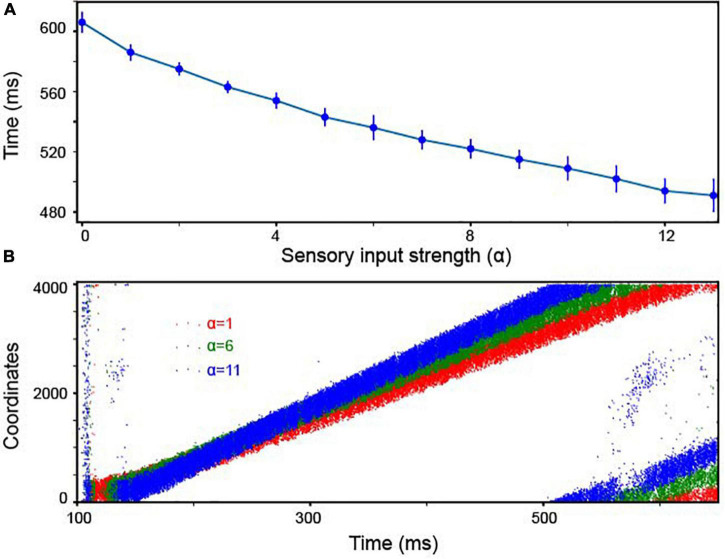
The continuous integrator was sensitive to the strength of the sensory inputs. **(A)** The travel time between consecutive Pyr neurons was inversely dependent on the strength of the sensory inputs; α represents the strength of the inputs to both Pyr and PV_1_ cells (Equation 3). In the experiment, we constructed 10 independent models, each of which was randomly constructed with the same rule and received independently created background noises. We display the mean values and standard deviations calculated from these 10 models. **(B)** Examples of propagating bump activity as a function of different input strength (i.e., different values of α in Equation 3).

Finally, how sensitive was our model to the strength of the stimulus inputs (i.e., the amount of sensory evidence)? Neurophysiological experiments have clearly shown that the rate of accumulation of the sensory evidence is positively correlated with the strength of the stimulus inputs. Further, this rate of accumulation is accompanied by a decrease in reaction time (Gold and Shadlen, 2007). To test whether our continuous integrator could account for this correlation between reaction time and stimulus inputs, we calculated how quickly activity traveled between adjacent Pyr neurons as a function of the strength (firing rate) of the sensory inputs, which is controlled by α in Equation 3. Indeed, as shown in Figure 5A, the travel time and α were inversely correlated. In other words, as strength of the sensory inputs increased, bump velocity also increased. This finding, in part, supports the correlation between behavioral reaction times and the strength of sensory evidence; examples of the propagation of bump activity through the network as a function of different values of α are shown in Figure 5B.

### Potential links to decision-making: The contribution of elective and exclusive connections between integrators and readout neurons

Sequential-sampling models, which can successfully account for perceptual decision-making, suggest that decisions can be made when the accumulated evidence reaches a decision-threshold (Ratcliff and Smith, 2004; Miller, 2015). For instance, race models assumes that evidence in support of one of two categorical choices is integrated independently and that a decision is reached whenever the accumulated evidence hits a decision-bound (Ratcliff and Smith, 2004; Miller, 2015). In principle, our lossless integrator can natively realize this accumulator model, as individual integrators can independently integrate evidence for available choices.

To address this possibility, we extend the model to perform a 2 alternative-forced-choice task, which is discussed below.

#### Gradient connections can implement relative thresholds for reaction-time decision-making

**FIGURE 6:**
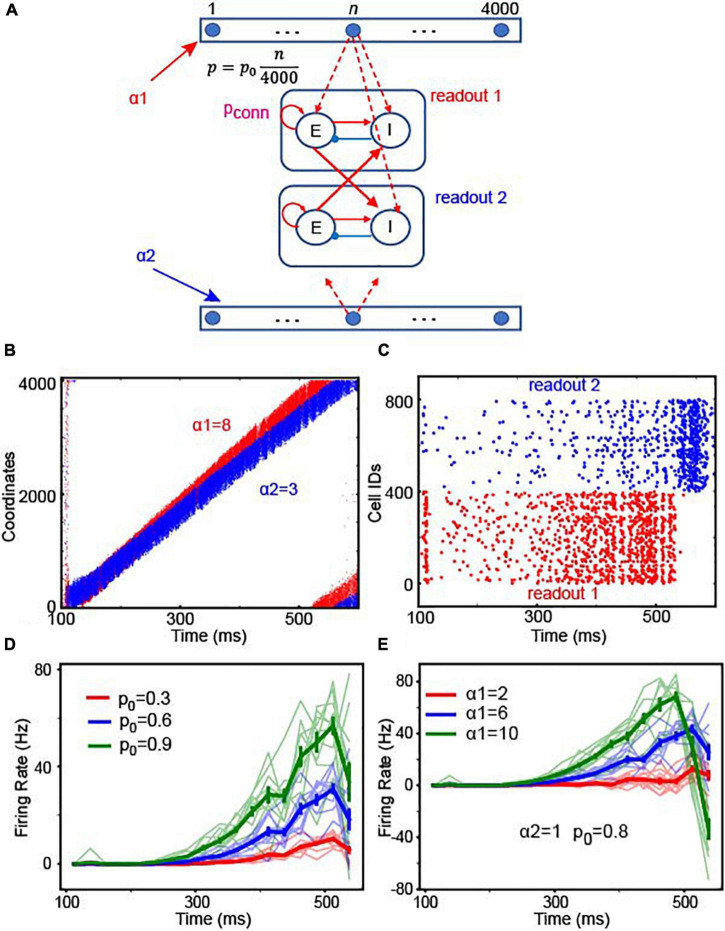
Readout schemes for decisions. **(A)** We assumed that there are two continuous integrators (top and bottom of the schematic) and that each Pyr neuron in each continuous integrator projected to excitatory neurons **(E)** in one of the two readout neuronal populations. The connection probability (*p* = $\frac{p_{0}}{4000}\,n$) increased, as the coordinate (n) of Pyr neurons increased. p_0_ is the maximal connection probability. In this simulation, both E and I neurons received 200-Hz external inputs *via* synapses whose strength was 1.3 pA. **(B)** Raster plot of the two integrators. The first and second integrators are represented in red and blue, respectively. Because the first integrator had stronger stimulus inputs (α1 = 8) than the second one (α2 = 3), the bump activity propagated faster in the first integrator than in the second. **(C)** Raster plots of the two populations of readout neurons, shown in red and blue, respectively. **(D)** Time course of firing rate difference between readout neurons depending on p_0_. In the experiments, we used 25 ms non-overlapping bins to estimate the time courses of population activity in 10 independent simulations in which α1 = 6, α2 = 1. In each simulation, we estimated the differences in the firing rates between readout neuron populations 1 and 2, which are shown in light red, green and blue lines. The thick red, green and blue lines represent the average firing rate over 10 experiments. The error bars denote the standard errors estimated from 10 experiments. The red, blue and green colors represent the results with p_0_ = 0.3, 0.6 and 0.9, respectively. **(E)** The time course of the firing rate difference between readout neurons depending on stimulus input strengths. We varied α1 in 10 experiments and estimated the difference in firing rates. As in panel **(D)**, the light color lines represent the results in the individual experiments, and the thick lines represent the average over 10 experiments. The error bars denote the standard errors estimated from 10 experiments.

For the reaction-time tasks, observers should be able to readout the amount of integrated evidence at any time. That is, if the brain relies on location-code NIs, it should be able to compare the locations of the bumps in the two integrators whenever necessary. This flexible comparison can be realized by connecting the integrator to readout neurons with “gradient connections.” In this gradient connection, the connection probability linearly increases as a function of the coordinates of integrator’s Pyr neurons. Pyr neurons in the integrator 1 projected to excitatory neurons in readout neuronal population 1 and inhibitory neurons in readout neuron population 2; integrator 2 is connected to readout neurons in an analogous manner (Figure 6A). This gradient connection is consistent with the experimentally observed connectivity (Perin et al., 2011) suggesting that connection probability decays over distance. The maximal connection probability p_0_ in the model can determine the overall number of connections between the integrator and readout neurons. Because integrator 1 received stronger sensory inputs (α1 = 8) than integrator 2 (α2 = 3), bump activity in the two integrators propagated at different speeds (Figure 6B). As seen in Figure 6C, readout neuron population 1 showed greater activity than population 2 until bump activity returned to the initial location due to the periodic boundary condition. Next, we further asked how the readout neuron neurons’ responses change depending on input strengths in two ways. First, we fixed the strength of sensory inputs (α1 = 6 and α2 = 1) and varied p_0_. Figure 6D shows the difference in the average firing rates between readout neuron populations. The light color lines show observations in 10 independent simulations, and the thick color lines, the average over 10 simulations. We found that the onset of readout neuron population 1 is negatively correlated with p_0_ (Figure 6D), suggesting that a faster decision can be made if stronger connections (i.e., higher p_0_) are established between location NI and readout neurons. Second, we fixed p_0_ and the strength of evidence to integrator 2 (α2 = 1) but varied the inputs to the integrator 1 (α1). In our 10 independent simulations (Figure 6E), we observed that decisions can be made faster if α1 − α2 (the difference in sensory evidence strength between the two choices) becomes stronger, which is consistent with the negative correlation between the reaction time and the ambiguity of sensory evidence (Gold and Shadlen, 2007).

#### Temporal profile of spiking activity in the readout neurons: Stepping vs. ramping

**FIGURE 7:**
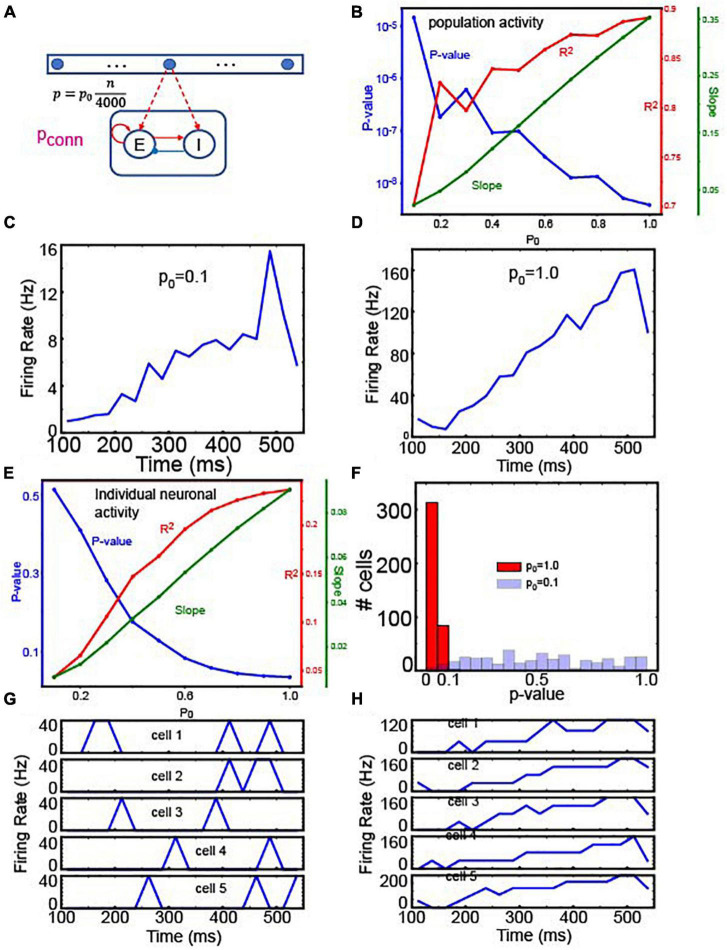
Readout neuron activity with gradient connections. **(A)** The structure of a single set of integrator and readout neurons. **(B)** Linear regression analysis of the average firing rate of 400 E readout neurons depending on p_0_. To see if the population activity ramps up, we used the linear regression analysis to test if the population activity is correlated with time. The positive slopes indicate the ramping activity. That is, this panel suggests that the population activity of readout neurons ramps in a wide range of p_0_. **(C)** Time course of population activity with p_0_ = 0.1 **(D)** the same as panel **(C)** but with p_0_ = 1.0. Panels **(C,D)** confirm the linear regression analysis in panel **(B)**. **(E)** Linear regression of individual neuron activity depending on p_0_. Unlike the analysis shown in panel **(B)**, we tested if individual neurons’ responses are correlated with time. In the panel, we showed the mean values from 400 readout neurons. This panel suggests that individual neurons’ responses are correlated with time only when p_0_ is sufficiently high. **(F)** Histograms of *p*-values from 400 readout neurons’ responses. In this panel, we compared two extreme cases, p_0_ = 0.1 and 1.0. As expected, most of the neurons’ responses are correlated with time when p_0_ = 1.0 **(G)**. Time course of individual neuronal activity with p_0_ = 0.1 **(H)**, the same as panel **(G)** but with p_0_ = 1.0.

The well-described ramping activity in area LIP strongly supports the existence of rate-code NIs (Roitman and Shadlen, 2002; Mazurek et al., 2003; Gold and Shadlen, 2007). However, recent studies have raised an alternative possibility that LIP activity does not smoothly ramp up but instead “jumps or steps” up to high-activity states during perceptual decisions (Miller and Katz, 2010; Latimer et al., 2015). Interestingly, even though individual neurons produce this stepping activity, the population activity still exhibits ramping activity. To shed some light on the nature of these two forms of LIP activity, we tested whether the readout neurons, which encode actual decision variables in our model, can reproduce either ramping or stepping activity by considering a single integrator and readout neuron population (Figure 7), for simplicity; this single integrator model replicates 100% coherence random-dot motion trials commonly used to investigate perceptual decision-making (Roitman and Shadlen, 2002; Mazurek et al., 2003).

To this end, we tested how well individual and population activities were correlated with time by utilizing the linear regression analysis. We first tested the correlations between population activities and time depending on p_0_. As shown in Figure 7B, population activities were significantly correlated with time, and the slope was positive, suggesting that population activities ramp up regardless of p_0_. The two examples at p_0_ = 0.1 and 1.0 confirmed that population activities ramped up (Figures 7C,D). On the other hand, individual neurons showed strikingly different behaviors depending on p_0_ (Figure 7E). When p_0_ was higher than 0.7, individual neuronal activity was significantly (*p* < 0.05) correlated with time. Notably, as p_0_ decreased, *p*-values became bigger. That is, individual cell activity was not significantly correlated with time, when p_0_ is low. To further test this notion, we compared the *p*-values of the regression analysis when p_0_ = 0.1 and when p_0_ = 1.0. When p_0_ = 1.0, the firing rates of most readout neurons (313 out of 400) were significantly correlated with time (*p* < 0.05), but when p_0_ = 0.1, only a fraction of neurons (6 out of 400) showed significant correlation (Figure 7F). The responses of 5 randomly chosen neurons confirmed that individual neurons showed transient activity (Figure 7G) when p_0_ = 0.1 but showed ramping activity when p_0_ = 1.0 (Figure 7H).

**FIGURE 8:**
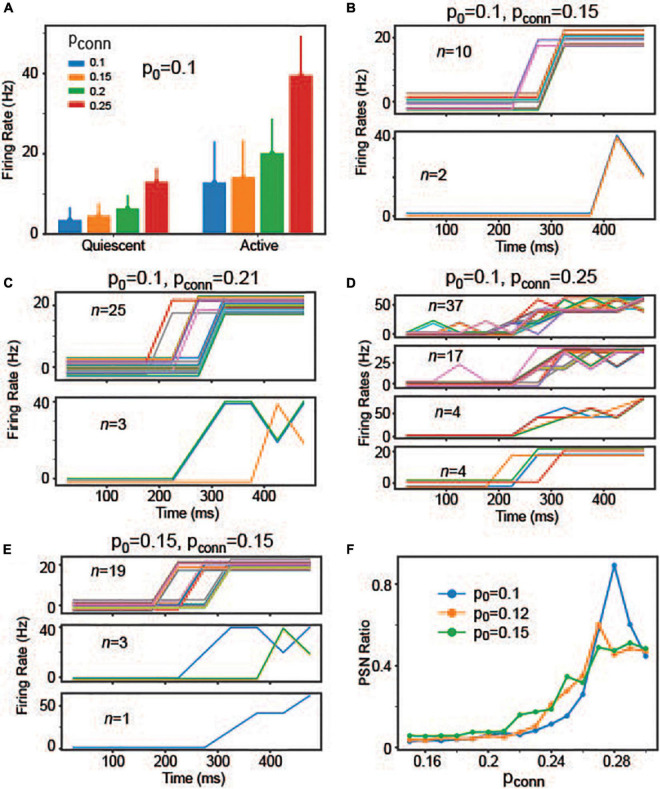
The individual readout neurons’ responses depending on p_0_ and p_conn_. **(A)** Individual neuron responses in the quiescent and active periods when p_0_ = 0.1 when p_conn_ = 0.1, 0.15, 0.2, and 0.25. **(B)** Firing rates of neurons that can be explained by the sigmoid function when p_0_ = 0.1 and p_conn_ = 0.15. For clarity, we split the neurons depending on the maximum firing rates. The neurons shown in the same panel share the same maximum rate. Individual neurons are displayed in different colors. Additionally, we added a random offset value (between –3 and 3) to each neuron’s firing rates to show all neurons more clearly. **(C)** The same as panel **(B)**, but p_0_ = 0.1 and p_conn_ = 0.21. **(D)** The same as panel **(B)**, but p_0_ = 0.1 and p_conn_ = 0.25. **(E)** The same as panel **(B)**, but p_0_ = 0.15 and p_conn_ = 0.15. **(F)** Ratio of neurons, whose responses can be explained by the sigmoid function. They are referred to as PSR neurons in the main text.

These results suggest that individual neurons’ responses are not necessarily correlated with population activities, which is the hallmark of the stepping activity model. Inspired by these results, we asked if readout neurons are capable of replicating stepping-like responses. In the stepping activity model (Durstewitz and Deco, 2008; Miller and Katz, 2010; Latimer et al., 2015), neurons switch rapidly between quiescent and active states, and their firing rates are stable (i.e., constant over time) in both quiescent and active states. To address this question, we first examined if readout neurons would undergo rate changes during decision-making (i.e., integration of evidence). Specifically, we estimated the time courses of firing rates using 25 ms time bins and then split them into quiescent and active periods. In the experiments, we estimated the mean firing rate over all time bins and determined the time (*T*) when the firing rate crosses the mean value for the first time. The quiescent period is between 100 ms and *T* when the firing rate crosses the mean value. The active period is between *T* and 550 ms. Figure 8A shows the changes in individual neurons’ firing rates between quiescent and active states depending on p_*conn*_ (i.e., the connection probability of recurrent connections within the readout neuron population), suggesting that individual neurons underwent rate changes during evidence integration. That is, the readout neurons may have binary states.

Next, we tested if the readout neurons abruptly switched from quiescent to active states, and if they have constant firing rates in both quiescent and active states. To this end, we estimated the time course of firing rates using 50 ms bins (to obtain smoother responses) and fitted them to the sigmoid function (Equation 2).


(2) $$S\,\left(x\right)=\frac{c}{1+e^{-a\,\left(x-b\right)}}+d$$

where a, b, c, and d are parameters optimized during curve-fitting.

After fitting individual neurons’ firing rates into the sigmoid function, we estimated R^2^ and selected neurons with *R*^2^ > = 0.85. When p_0_ = 0.1 and P_*conn*_ = 0.15, 12 readout neurons showed stepping-like responses (Figure 8B). The number of neurons, showing stepping-like responses, grew when p_*conn*_ was increased to 0.21 (Figure 8C). When p_*conn*_ was strengthened further (for instance, p_*conn*_ = 0.25), some neurons showed multiple activity states (rather than binary) or the transitions from quiescent to active states took long (Figure 8D). That is, some neurons’ responses morphed into ramping-like responses. Interestingly, we found that the number of potential stepping-response (PSR) neurons increased when p_0_ increased (Figure 8E). To better understand how p_0_ and p_*conn*_ influence readout neurons’ response patterns, we estimated the number of neurons with R^2^ higher than 0.85 (i.e., PSR neurons that can be explained well by the sigmoid function). We made two observations (Figure 8F). First, the number of PSR neurons initially increased as p_*conn*_ increased but started decreasing after p_*conn*_ ∼ 0.28. Indeed, when the p_*conn*_ was too high, most of neurons’ responses were ramping. Second, p_0_ increased the number of PSR neurons, when p_*conn*_ was lower than 0.25. These results raised the possibility that decision neurons could show either stepping or ramping activities depending on the strength of evidence (modeled with p_0_ in the model) and recurrent interactions between them (modeled with p_*conn*_ in the model).

## Discussion

Perceptual decision-making relies on the accumulation of sensory evidence (i.e., decision-variables) that is extracted from ambiguous sensory stimuli (LaBerge, 1962; Ratcliff, 1978; Roitman and Shadlen, 2002; Mazurek et al., 2003; Ratcliff and Smith, 2004; Smith and Ratcliff, 2004; Miller, 2015). It is generally thought that perceptual decision-making is instantiated through rate-code neural integrators (NIs), which are based on recurrent inputs to compensate for the leak currents (Goldman et al., 2009; Wang, 2012). However, the degree to which rate-code NIs can explain perceptual decision-making can be limited. For example, rate-code NIs become unstable when there is a temporal gap in the flow of incoming sensory evidence (Figure 2), whereas behavioral studies indicate that participants act as “perfect/lossless” integrators and are not affected by these temporal gaps (Kiani et al., 2013; Liu et al., 2015).

How then can the brain make reliable decisions even with temporal gaps? We propose that the cortex can readily use the location of bump activity to represent the amount of presented sensory evidence (Skaggs et al., 1995; Song and Wang, 2005; see below). In our simulations, bump activity in the integrator progressed through the network when sensory inputs were provided but stayed at the same location in the absence of sensory information. The location of the bump was stable due to the inhibition of SST cells (Figures 3, 4). This indicates that our integrator, unlike traditional rate-code NIs, can account for the robustness of perceptual decision-making during temporal gaps in sensory evidence.

### Comparison to other location code neural integrators

In terms of function, our model reproduces the findings of previously reported location-code NIs, which modeled head-direction neurons encoding the direction of an animal’s head relative to its body and independent of its location in the environment (Song and Wang, 2005). However, the underlying mechanisms between our NI and previously described ones are quite distinct.

In previous location-code NIs, the shift in the location of bump activity was realized by so-called “rotation” neurons, which employed either strictly excitatory neurons (Skaggs et al., 1995) or strictly inhibitory neurons (Song and Wang, 2005); these rotation neurons are located in the portion of the thalamus that receives inputs from the vestibular system. In contrast, we found that a cortical circuit, which consisted of excitatory pyramidal neurons and different types of inhibitory interneurons, can readily implement a location-code NI.

More specifically, two common inhibitory cortical neurons (Rudy et al., 2011)—PV and SST interneurons—made distinct contributions to this operation. PV neurons, which provided nonspecific feedback inhibition to pyramidal neurons (Ma et al., 2010; Bock et al., 2011), ensured that bump activity existed only at a single location. On the other hand, SST neurons mediated lateral inhibition and transformed the network into an effective attractor network capable of maintaining accumulated evidence even during temporal gaps in sensory information (Figures 3C, 4G). We note that this theoretical finding is consistent with the empirical finding that SST cells are selectively activated during a delay period when a stimulus is removed and an animal needs to remember task-relevant information (Kim et al., 2016). In contrast to the role that interneurons and their inhibitory synapses played in our network model, depressing excitatory synapses made bump activity propagate through the network (Figure 3D). Together, our simulation results suggest that neurons and synapses in the neocortex are indeed suitable for controlling and maintaining the propagation of bump activity.

### Connections to the rate-code neural integrators

Earlier theoretical and computational studies proposed the rate-code Nis that are robust to the imbalance between leak currents and feedbacks (see Koulakov et al., 2002; Goldman et al., 2003; Cain et al., 2013). That is, our location-code NI is similar to these robust integrators in terms of functions. However, the aim of our study is to gain insights into the recently proposed stepping activity model (Latimer et al., 2015; Zoltowski et al., 2019) and its potential links to the ramping activity. In our model, ramping or stepping activity can emerge depending on afferent inputs from a location-code NI. Dense gradient connections (i.e., high p_0_) induce the ramping activity, whereas sparse gradient connections (i.e., low p_0_) induce the stepping activity, raising the possibility that the two seemingly different models could represent the two faces of the same coin.

Further, our simulation results suggest that the recurrent readout neuron populations can convert accumulated evidence in the location-code NI into ramping or stepping activities. That is, the location-code NI, providing a “neural memory buffer,” may be complementary to the rate-code NI and then enable to the brain retain accumulated evidence during the temporal gap. Then, the question is, why do we detect ramping activity more frequently than stepping activity? This may be because the memory buffer provided by the location-code NI is not always necessary. If the temporal gap rarely occurs, the brain need not maintain the memory buffer (i.e., the location-code NI). Instead, the rate-code NI alone can sufficiently perform reliable decision-making most of the time. Notably, the common random dot motion protocol does not contain temporal gaps.

### Empirical evidence for location-based neural integrators relying on bump activity

Sequential activation, consistent with bump activity propagation in our model, has been observed in multiple brain regions (Tang et al., 2008; Pulvermuller and Shtyrov, 2009) including the visual cortex (Ikegaya et al., 2004; Sato et al., 2012; Xu et al., 2012), parietal cortex (Harvey et al., 2012) and frontal cortex (Seidemann et al., 1996). Notably, Harvey et al. (2012) found that posterior parietal cortex neurons were sequentially activated during decision-making, raising the possibility that the location-code NI can exist in cortical regions like area LIP. That is, it is plausible that both location-code NIs and readout neurons coexist in area LIP, in which both stepping and ramping activities have been observed. It should be noted that the gradient connections in our model, which are necessary to account for stepping and ramping activities, are consistent with experimental findings (Perin et al., 2011) that the connection probability decreased as the distance between neurons.

### Limitation of our model and concluding remarks

In this study, we only considered a 2-choice task, but it should be noted that the location-code NI can also be used for multiple-choice tasks. If multiple choices are available, the evidence supporting each choice could be tracked by an independent location-code NI. When the decisions are required, the readout neurons could determine the best choice using the winner-take-all mechanism.

While the determination of the exact mechanisms behind any cognitive functions remains difficult, we would like to underscore that our model demonstrates that cortical circuits can natively switch between two seemingly distinct states, the stable steady state (e.g., bump activity maintenance) and the sequential activation state (e.g., bump activity propagation). We are not arguing that location-code NIs preclude the existence of rate-code Nis in neural systems. As they have distinct pros and cons, we speculate that location- and rate-code NIs are rather complementary and can be selected depending on cognitive demands. We also note (1) that, *to the best of our knowledge*, there is no direct evidence supporting the location-code NI associated with perceptual decision-making and (2) that our model has a complex structure with fine-tuned parameters, and thus it remains unclear if our model is physiologically realizable. We will further study the properties of the newly proposed location-code NI to address these limitations.

## Materials and methods

In this study, we developed lossless neural integrators, which were implemented within the NEST environment (Gewaltig and Diesmann, 2007), a peer-reviewed, freely available simulation package. All neurons in the model were leaky integrate-and-fire (LIF) neurons. The excitatory and inhibitory neurons within an integrator formed excitatory and inhibitory connections onto a set of “target” neurons. All integrator neurons and target neurons had identical internal dynamics; specifically, each presynaptic spike induced an abrupt increase in a neuron’s membrane potential that decayed exponentially. These neurons were implemented using the native NEST model iaf_psc_exp (Gewaltig and Diesmann, 2007). Table 1 shows the exact parameters used for the neurons and synapses in both neural integrators.

**TABLE 1:** Neural parameters for neurons and synapses.

Neuronal Parameters		Synaptic parameters	
C (membrane capacitance)	1 pF	τ_*syn*_	2.0 ms
V_*th*_ (spike threshold)	20 mV	Delay	1.5
τ_*m*_ (membrane time constant)	20 ms	U	0.2
E_*L*_ (resting membrane potential)	0 mV	τ_*ref*_	200 ms for discrete integrator 500 ms for continuous integrator
V_*reset*_ (reset after spiking)	0 mV		

When a spike arrived, the membrane potential instantly jumped to a new value, which was determined by its capacitance (C) and time constant (τ_*m*_). When the membrane potential was higher than the spike threshold, the membrane potential was reset (V_*reset*_). Without any external input, the membrane potential relaxed back its the resting membrane potentials (E_*L*_). Synaptic events decayed exponentially with a 2-ms time constant (τ_*syn*_). All synapses had a 1.5-ms delay unless otherwise stated; the only exception is given in Table 2. For depressing synapses, we selected the parameters (U and τ_*ref*_) given below.

### The structure of the discrete integrator

The structure of the discrete integrator is summarized in Figures 1A,B. As seen in Figure 1A, the discrete integrator consisted of 19 different neuronal populations. 17 of these neuronal populations contained 400 pyramidal (Pyr) and 16 somatostatin (SST) model neurons. Within each of these 17 populations, Pyr neurons formed excitatory synapses with both Pyr and SST neurons. These 17 populations were topographically organized: Pyr neurons within a population had unidirectional excitatory connections with the adjacent population (e.g., population 2 projected to population 3 but not back to population 1). We had a periodic boundary condition in which the (last) population 17 connected to the (first) population 1 (see Figure 1B). In contrast, SST neurons formed inhibitory connections with Pyr neurons in all of the other populations. Recurrent connections between Pyr neurons within a particular population had depressing synapses (Markram et al., 1998; Reyes et al., 1998; Fuhrmann et al., 2002; Petersen, 2002; Cheetham and Fox, 2010; Lefort and Petersen, 2017), but all of the other synaptic connections were static. We implemented these depressing synapses using the Tsodyks-Markram model included in the NEST distribution (Table 1).

The two remaining populations each had 1,088 parvalbumin (PV) neurons. All of the Pyr neurons had excitatory connections with the PV neurons in one population (PV_1_) but not with those in the second PV population (PV_2_). Both PV_1_ and PV_2_ neurons formed non-specific inhibitory connections with Pyr and SST neurons; see Table 2 for the connection probability. These two PV populations simulated feedback and feedforward inhibition between Pyr neurons.

**TABLE 2:** The parameters of the discrete integrator.

	Total number	Background inputs (Hz)	Stimulus input (Hz; sustained)
Pyr	6,800	2,800	2,000
PV_1_	1,088	4,500	2,000
PV_2_	1,088	N/A	2,000
SST	544	3,200	N/A
**Connectivity within populations (connection probability, strength in pA)**			
Pyr → Pyr	(1.0, 1.8)	Pyr → SST	(0.4, 0.96)
PV_1_ → PV_1_	(0.3, −0.72)	PV_1_ → PV_1_	(0.1, −0.72)
**Connectivity across populations (connection probability, strength in pA)**			
Pyr → Pyr	(0.2, 0.12) *delay 10 ms	PV_2_ → SST	(1.0, −6.0)
Pyr → PV_1_	(0.2, 0.12)	SST → Pyr	(1.0, −4.8)
PV_1_ → Pyr	(0.2, −1.08)	SST → PV_1_	(0.3, −0.6)
PV_1_ → SST	(0.3, −0.6)		
**Connection strength for background and stimulus inputs in pA**			
Pyr	0.12	PV_2_	0.36
PV_1_	0.12	SST	0.12
**Onset stimulus input**			
Target	Pyr neurons in population 1	Firing rate	1,000 Hz

We connected populations by specifying connection probabilities and synaptic connection strengths. The first value in the parentheses is the connection probability. The connection strengths followed Gaussian distributions. The mean values of these distributions are the second value in the parentheses, and the standard deviations were 10% of the mean. The excitatory and inhibitory connections could not be less than or greater than 0, respectively; when they violated this condition, we set them to 0. We note that the connection strengths greatly vary depending on the pairs of neurons. For example, the inhibitory connections from PV_2_ to SST are 10 times stronger than those from PV_1_ to SST.

### The structure of the continuous integrator

The continuous integrator was composed of a population of Pyr neurons, two PV populations (PV_1_ and PV_2_), and two populations of SST neurons (SST_1_ and SST_2_); see Figure 1C. Table 3 lists the parameters of these neuronal populations; see Supplementary Figure 2 for visual presentation of synaptic connections between neuron populations. In this network, 4,000 Pyr, SST_1_ and SST_2_ neurons were distributed in a circular lattice, each of which had unique coordinate between 1 and 4,000. We arbitrarily set the coordinates to increase in the clockwise direction. The neuronal numbers were arbitrary and were not constrained by the ratio of excitatory to inhibitory neurons, which is roughly 4:1. It should be noted that it is straightforward to extend this network model to include more excitatory neurons. For example, instead of a single Pyr neuron at each coordinate, a small population of Pyr neurons at each coordinate can be instantiated without changing any of the details of the network structure.

**TABLE 3:** The parameters of the continuous integrator.

	Total number	Background inputs (Hz)	Stimulus input (Hz)
Pyr	4,000	3,850	4,800
PV_1_	1,000	3,850	1,200
PV_2_	1,000	3,000	1,200
SST_1_	4,000	2,000	N/A
SST_2_	4,000	2,000	N/A
**Connectivity (Number of presynaptic neurons, strength in pA)**			
Pyr → Pyr	(400, 0.52)	PV_1_ → SST_1_	(150, −0.78)
Pyr → PV_1_	(400, 0.52)	PV_2_ → SST_1_	(1,000, −0.78)
Pyr → SST_1_	(1, 11.7)	SST_1_ → Pyr	(3,600, −0.78)
Pyr → SST_2_	(1, 11.7)	SST_1_ → PV_1_	(1,200, −0.78)
PV_1_ → Pyr	(160, −1.87)	SST_2_ → Pyr	(400, −0.78)
PV_1_ → PV_1_	(160, −0.78)		

Due to the lack of population structure, we connected neurons by specifying the number of presynaptic neurons to each neuron type. The frequency of stimulus inputs given below is the default value used unless stated otherwise; see also Equation 3. The first value is the number of presynaptic neurons, and the second value is the connection strength in pA. The excitatory and inhibitory connections could not be less than or greater than 0, respectively; when they violated this condition, we set them to 0. The background inputs to all neurons in the continuous integrator are mediated by synapses whose strength are 0.13 pA.

Pyr neurons were mutually connected, *via* excitatory connections, to their neighboring Pyr neurons when the difference between their coordinates was ≤± 200, which is equivalent to a distance-dependent connection probability (Perin et al., 2011). These connections were established with a periodic boundary condition: Pyr neuron 4,000 and Pyr neuron 1 were mutually connected.

Pyr neurons interacted with the PV_1_, SST_1_, and SST_2_ populations in distinct ways. First, the pattern of connectivity between the Pyr and PV_1_ populations was randomly generated. Second, a Pyr neuron projected only to those SST_1_ and SST_2_ neurons that had the same coordinates (i.e., a one-to-one topographic mapping). The connection strength was designed to be just strong enough for a single Pyr “spike” to cause a SST_1_ or SST_2_ neuron to fire (Table 3), like a single layer-5 pyramidal-neuron spike can induce SST-expressing Martinotti neurons to fire (Silberberg and Markram, 2007). Finally, SST_1_ and SST_2_ neurons also had inhibitory connections with Pyr neurons but had different connectivity rules. SST_1_ neurons formed connections only with those Pyr neurons in which the SST_2_-and-Pyr difference was ≥200. In contrast, SST_2_ neurons formed connections only with those Pyr neurons with lower coordinate values.

Other important model details are that PV_2_ neurons randomly inhibited SST_1_ neurons; the connection probability is shown in Table 3. Further, the PV_1_ and PV_2_ populations were independent of this circular lattice (see Figure 1C). In our continuous integrator, all excitatory synapses between Pyr neurons were depressing, whereas all inhibitory synapses were static.

### External inputs for both integrators

The excitability of each neuron depended on the sum of its synaptic inputs from all of the other neurons in the network and from external inputs. Tables 2, 3 show the neuron-specific rates of these external inputs, which were modeled with Poisson spike trains. In the model, there were “background” and “stimulus inputs” (i.e., sensory information). Background inputs were independent of stimulus presentations and mimicked afferent inputs from other cortex (Potjans and Diesmann, 2014). Stimulus inputs had both “transient” and “sustained” modes of activity. The transient mode represented the transient onsets of neural activity that have been observed in the sensory systems including retina, lateral geniculate nucleus and cortex (Cleland et al., 1971; De Valois et al., 2000; de la Rocha et al., 2008; Piscopo et al., 2013). We assumed that this transient activity helped to ensure that bump activity was always initiated at the same location in the network. Transient inputs (duration: 100 ms) were introduced to the first 400 and 100 Pyr neurons in the discrete and continuous integrators, respectively. In contrast, the sustained sensory inputs formed projections with all Pyr, PV_1_, and PV_2_ neurons during the entire stimulus. The frequency (I_*sustained*_) of the sensory inputs to PV_1_ neurons is given in Equation 3, and Pyr neurons received sensory inputs equivalent to 4 × I_*sustained*_.


(3) $$I_{s\,u\,s\,t\,a\,i\,n\,e\,d}=400+\alpha \times 100\,\left(H\,z\right)$$

### Traveling time for the bump

Using the continuous integrator, we tested the relationship between the propagation speed of the bump and the strength of the sensory input by calculating the time course of the last 400 Pyr neurons (i.e., those with 400 highest coordinates). Specifically, we generated an event-related spike histogram using non-overlapping 10-ms bins of spiking data. “Traveling time of the bump” was defined as the time, relative to stimulus onset, when the number of spikes in a single bin exceeds the sum of the mean plus two standard deviations of the number of spikes during the simulation period.

## Data availability statement

The raw data supporting the conclusions of this article will be made available by the authors, without undue reservation.

## Author contributions

JL, JT, SV, and YC designed research and wrote the manuscript. JL performed the research and analyzed the data. All authors contributed to the article and approved the submitted version.
