# Supplementary material for: Cortical circuit-based lossless neural integrator for perceptual decision-making: A computational modeling study
Source: Front Comput Neurosci. 2022 Nov 3;16:979830. doi: 10.3389/fncom.2022.979830 (PMC9669970; doi:10.3389/fncom.2022.979830)
Supplement: Supplementary file 1 [file Data_Sheet_1.docx]

Supplementary Material

# Supplementary Figures


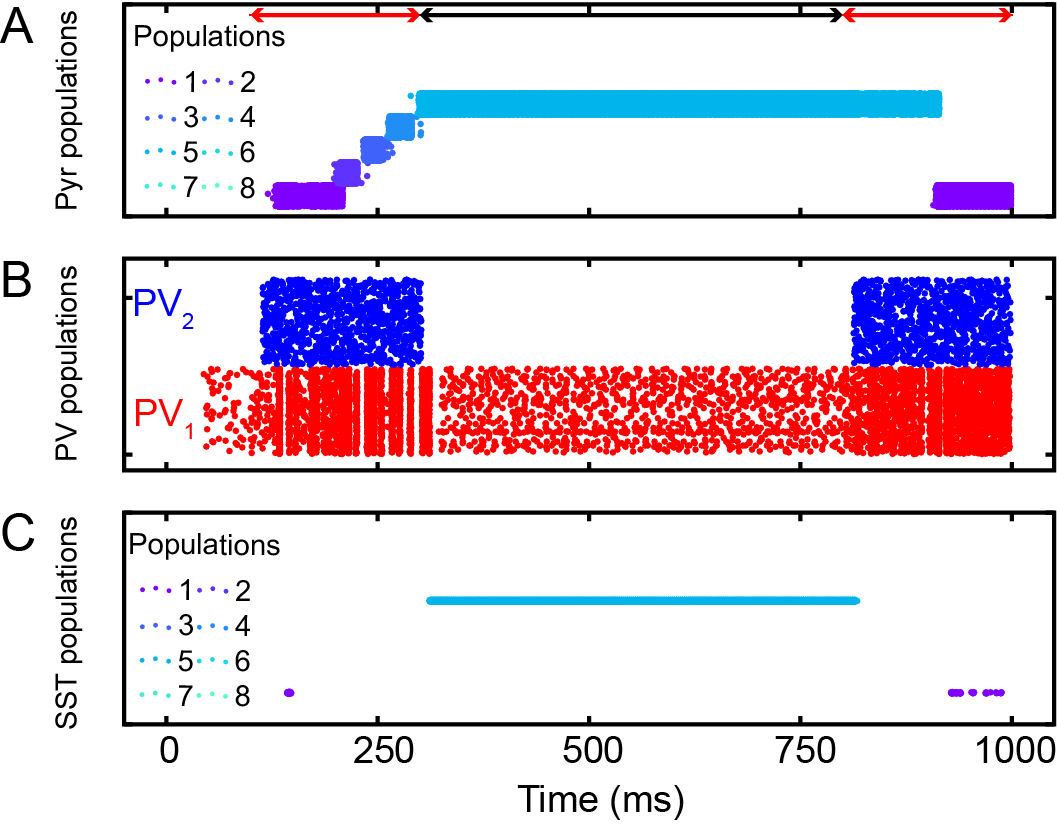


**Supplemental Figure 1: The responses of populations without inhibition of PV_1_ to Pyr_1_ populations during the temporal gap.** We repeated the same experiment shown in Fig. 3, but removed inhibition from PV1 to Pyr1 populations at 500 ms. That is, the first half of the responses are identical to those shown in Fig. 3. But the last half are different. As expected, when stimulus inputs were reintroduced, population 1 was reactivated by the transient inputs; this destroyed the accumulated information. Conversely, the feedback inhibition of PV_1_ neurons ensures the network accurately encodes the evidence accumulated.


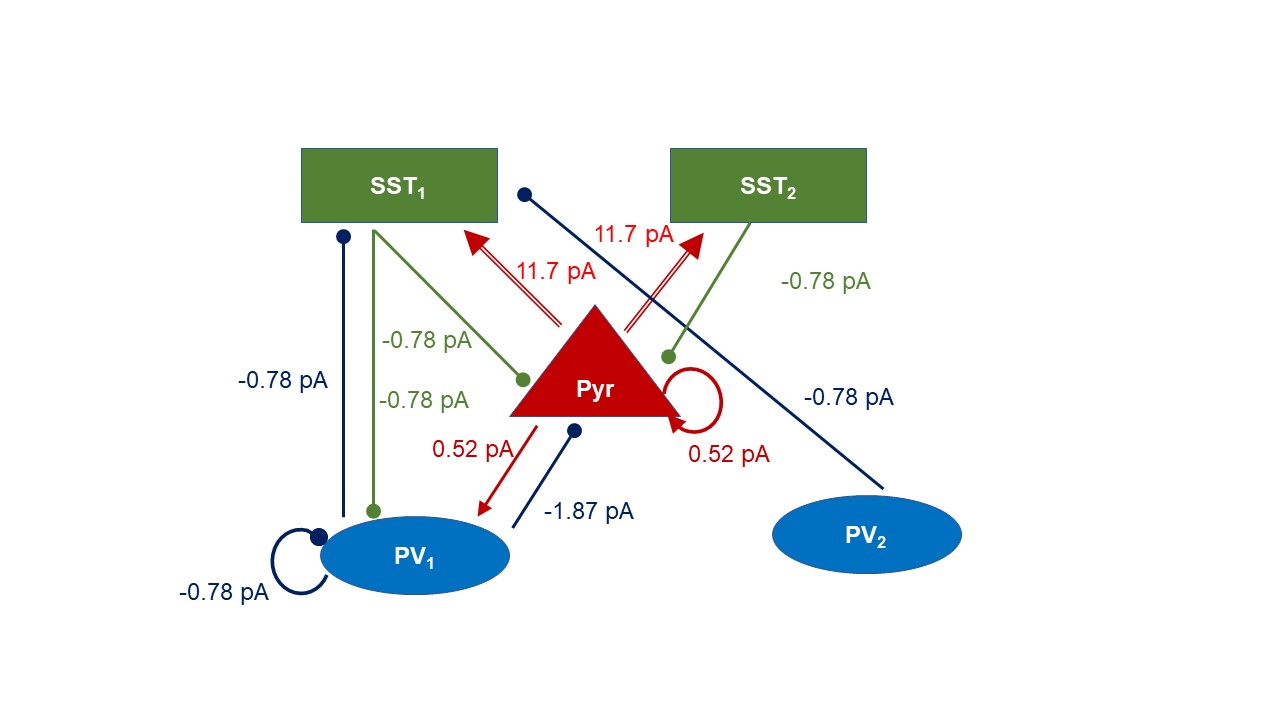


**Supplemental Figure 2: The diagram of the synaptic connections in the continuous integrator.** The excitatory connections are shown in red arrows, the inhibitory connections from PV neurons are shown in blue arrows, and the inhibitory connections from SST neurons are shown in green arrows.
